# Prevalence and Predictors of Somatization in Peruvian Undergraduate Students during the COVID-19 Pandemic

**DOI:** 10.3390/ijerph192315576

**Published:** 2022-11-24

**Authors:** Angel Christopher Zegarra-López, Giancarlo Luna-Victoria, Daniella Romero-Montenegro, Brian Florentino-Santisteban, Diego Eduardo Prieto-Molinari, Mitchell Montoya-Cuadrao

**Affiliations:** 1 Faculty of Psychology, Universidad de Lima, Lima 15023, Peru; 2 Grupo de Investigación en Psicología, Bienestar y Sociedad, Instituto de Investigación Científica, Universidad de Lima, Lima 15023, Peru

**Keywords:** somatization, mental health, Peru, COVID-19, depression, anxiety, stress, resilience, self-efficacy, well-being, undergraduate students

## Abstract

The COVID-19 pandemic had a strong impact on mental health. Multiple studies report the alarming prevalence of depression, anxiety, and stress-related conditions due to the lockdown measures. Nevertheless, somatization has been an overlooked topic in current literature despite its strong relationship with most mental health conditions. The aim of this study was to describe the prevalence of somatic symptoms and their associated factors in a sample of 3218 undergraduate students from Lima, Peru. A cross-sectional design was carried out. The prevalence of somatic symptoms was measured with the PHQ-15. As predictors of somatic symptom severity, we included psychopathological (depression, anxiety, and stress), psychological (perceived social support, resilience, satisfaction with life, and academic self-efficacy), and sociodemographic (e.g., age, sex, employment status, relationship status, daily hours of sleep) variables. A generalized linear model from a binomial family and a logit link function were applied based on a Factor Score Regression approach, with half of the sample presenting moderate-to-severe somatic symptoms. Anxiety was the strongest predictor of somatic symptom severity, followed by academic self-efficacy. Significant differences were found regarding sex, relationship status, daily hours of sleep and COVID-19 risk-related variables. In conclusion, interventions on reducing anxiety and promoting academic self-efficacy may have a stronger impact on somatic symptom severity and should focus on more vulnerable specific demographic groups such as females.

## 1. Introduction

The sudden outbreak of the Severe Acute Respiratory Syndrome Coronavirus 2 (SARS-CoV-2) in Wuhan, China, in 2019 and its subsequent declaration as a pandemic in March 2020 forced most governments across the world to impose strict measures such as the closure of nonessential establishments, travel restrictions, social isolation, and compulsory lockdowns [1]. Consequently, there has been a strong impact on multiple sectors, such as healthcare systems, education, industries, and social dynamics [2]. Mental health has been one of the most concerning consequences of the COVID-19 pandemic and confinement measures [3,4]. Several studies report the prevalence of mental health conditions in the general population and in specific age groups in different contexts [5,6,7,8].

According to research, young adults are one of the most affected groups due to the nature of their developmental stage, along with stressors such as fear of infection, social distancing and confinement, suspension of regular academic activities, and global financial recessions [6]. As a result, several mental health-related conditions such as depression, anxiety and stress have been reported in youth populations [7]. In this sense, a constant experimentation of negative emotions can lead to somatic symptoms which may become a source of discomfort and have a strong impact on mental and physical health [9,10]. The manifestation of somatic symptoms without an explicit pathophysiological explanation are amidst the most common problems in health services, and they are usually shown as fatigue, lack of energy, sleep problems and back, head, abdominal, and chest pain [11,12]. In addition, somatic symptoms may hinder results from mental health treatments, reduce quality of life and cause general functional impairments [13].

Additionally, the COVID-19 pandemic has been related to an increased risk of suffering from somatic symptoms due to its impact on the central nervous system or secondary effects from treatments, along with mental health conditions which may increase the chance of developing somatic symptom disorders [14]. Nevertheless, the somatic syndrome has been an overlooked topic on the psychological impact of COVID-19, even though past pandemics and infectious diseases have been related to somatic disorders [15]. For this reason, the aim of this study was to describe the prevalence of somatic symptoms in a sample of young adult university students. Only a few studies have addressed the prevalence of somatic symptoms on this specific population; still, they recognized its importance due to the constant academic stressors that undergraduate students go through, and an increased report of mental health conditions related to the pandemic [9,16].

To further understand the somatic symptom severity, we aimed to analyze potential sociodemographic-, psychological-, and psychopathological-associated factors. The reason for including sociodemographic characteristics such as sex, age, marital status, and employment status is because they have shown to be related to persisting somatic symptoms [17]. Psychological variables such as resilience, academic self-efficacy, perceived social support and satisfaction with life were included, since multiple studies show that they represent protective factors against mental health conditions during the pandemic [18,19,20,21,22]. Measures of mental health-related conditions such as depression, anxiety and stress were included because of the strong evidence regarding their close relationship with somatic symptoms [23,24], even in lockdown settings [25]. As most relationship studies have focused on fewer factors, we aimed to simultaneously analyze multiple variables on a single model to identify the most relevant predictors of somatic symptom severity.

## 2. Materials and Methods

### 2.1. Study Design and Subjects

A cross-sectional study was designed based on information available on secondary data obtained from an online survey carried out by a private university from Lima, Peru. The data collection process was based on an online survey, made available for students enrolled during 2020; their participation was strictly voluntary and required an expressed consent. The survey included measures of somatic symptoms along with sociodemographic questions and other psychological scales for variables such as resilience, social support, positive and negative effects, depression, anxiety and stress, and satisfaction with life. After data curation procedures, a sample of 3218 respondents was included with ages ranging from 18 to 24 (Mean = 20.28; Standard Deviation = 1.66) and composed of 31.95% men and 68.05% women. As stated by the Code of Ethics and Deontology of the institution Colegio de Psicólogos del Perú, the study was proposed based on the national and international normativity regarding research on humans and was approved by the Research and Ethics Committee of the Faculty of Psychology of the Universidad de Lima.

### 2.2. Measures

#### 2.2.1. Somatization

The prevalence of somatic symptoms was measured with the Patient Health Questionnaire-15 (PHQ-15) [26], a self-report subscale based on the Primary Care Evaluation of Mental Disorders (PRIME-MD). The PHQ-15 aims to identify 15 somatic symptoms, each presented on a 3-point response scale from Not bothered at all (0) to Bothered a lot (2). Total scores of 5, 10, and 15 may be used as thresholds to classify somatic symptom severity as none (0–4), low (5–9), medium (10–14), or high (≥15), respectively. We decided to exclude two items: item 4 on sexual pain and problems for several reasons previously explained in the literature [27], and item 11 on menstrual problems, since it only applied to female participants [28]. Assuming a unidimensional congeneric measurement model, we estimated a reliability coefficient of ω = 0.82.

#### 2.2.2. Resilience

Resilience was operationalized with a short 10-item version of the Connor–Davidson Resilience Scale (CD RISC-10) [29]. In this instrument, each item is presented on a 5-point Likert-type response scale. We estimated a reliability coefficient of ω = 0.89 on a unidimensional model χ^2^(35) = 374.87, CFI = 0.960, RMSEA = 0.055 (90% CI: 0.050–0.060), SRMR = 0.026.

#### 2.2.3. Perceived Social Support

Perceived social support was measured using the Multidimensional Scale of Perceived Social Support (MSPSS) [30]. This is a 12-item scale which measures the perceived support of family, friends, and a significant other using a 6-point Likert response scale. The estimated reliability coefficients were ω = 0.87, ω = 0.90, and ω = 0.94, respectively, on the theoretical 3-factor model χ^2^(51) = 942.50, CFI = 0.918, RMSEA = 0.074 (90% CI: 0.070–0.078), SRMR = 0.035.

#### 2.2.4. Depression, Anxiety, and Stress

Mental health conditions were assessed through the Depression, Anxiety, and Stress Scales (DASS-21) [31]. Each subscale is composed of 7 items presented in a 4-point response scale. Reliability coefficients for the depression (ω = 0.90), anxiety (ω = 0.83), and stress (ω = 0.85) subscales were adequate in a multidimensional correlated 3-factor model: χ^2^(186) = 3126.20, CFI = 0.880, RMSEA = 0.070 (90% CI: 0.068–0.072), SRMR = 0.044.

#### 2.2.5. Satisfaction with Life

The Satisfaction with Life Scale (SWLS) [32] was included in the design as a short measure of general satisfaction of an individual towards their own life. The scale has 5 items in a 7-point response scale. We estimated a reliability coefficient of ω = 0.82 on a unidimensional model: χ^2^(5) = 53.71, CFI = 0.986, RMSEA = 0.055 (90% CI: 0.042–0.069), SRMR = 0.018.

#### 2.2.6. Academic Self-Efficacy

Academic self-efficacy was measured using the Perceived Self-Efficacy Scale in Academic Situations (EAPESA) [33]. We used a 9-item version of the scale previously tested on Peruvian university students. The items are presented with a 4-point Likert response scale from Never to Always. We found strong reliability evidence (ω = 0.93) on a well-adjusted unidimensional model: χ^2^(27) = 646.03, CFI = 0.991, RMSEA = 0.084 (90% CI: 0.079–0.090), SRMR = 0.026.

#### 2.2.7. Sociodemographic Variables

The sociodemographic variables included were age, sex, body mass index, relationship status, employment status, daily hours of sleep, high risk for COVID-19, having a relative who was diagnosed with COVID-19 (i.e., diagnosed with previous chronic illnesses such as asthma, diabetes, heart disease, cancer, AIDS, and others), and having a relative whose work exposes them to the disease. The latter of these variables may help understand how fear of contagion in close circles, whether it be a close relative or one who resides with the individual, may turn into stressors that further the somatic symptoms occurrence.

### 2.3. Analysis

Prevalence rates were estimated for each individual item on the whole sample. Then, the percentage of individuals on each somatic symptom severity category was calculated for all participants in each relevant sociodemographic group. To analyze factors associated with somatic symptoms, we used a Factor Score Regression (FSR) approach, which divides the main analysis in two stages. The first stage started off by fitting independent measurement models for the CD-RISC, MSPSS, DASS-21, SWLS and EAPESA using Confirmatory Factor Analysis based on polychoric correlation matrices and the WLSMV estimator as an optimal approach while modeling ordinal data [34]. All models were assessed through the Comparative Fit Index (CFI), Root Mean Squared Error of Approximation (RMSEA) and Standardized Root Mean Square Residual (SRMR). CFI ≥ 0.95, RMSEA ≤ 0.05 and SRMR ≤ 0.06 denoted an excellent fit, whereas CFI ≥ 0.90, RMSEA ≤ 0.08 and SRMR ≤ 0.08 pointed to a reasonable fit [35]; nevertheless, we expected a higher RMSEA due to our large sample size and the choice of the WLSMV estimator. Reliability estimates were also obtained through the Coefficient Omega with the procedure suggested by Green and Yang [36]. The second stage consisted of estimating factor scores based on the proposed measurement models, which serve as indicators of the relative location of each individual on the corresponding latent trait [37]. Then, a generalized linear model from a binomial family and a logit link function were carried out [38], considering somatic symptom severity as a dichotomous dependent variable with levels none to low (0), and moderate to severe (1). Factor scores were introduced as independent variables along with other sociodemographic characteristics from individuals. All analyses were programmed in R (version 4.1.2) using mainly the lavaan (version 0.6-9) and semTools (version 0.5-5) packages.

## 3. Results

### 3.1. Prevalence of Somatic Symtpoms

**Figure 1:**
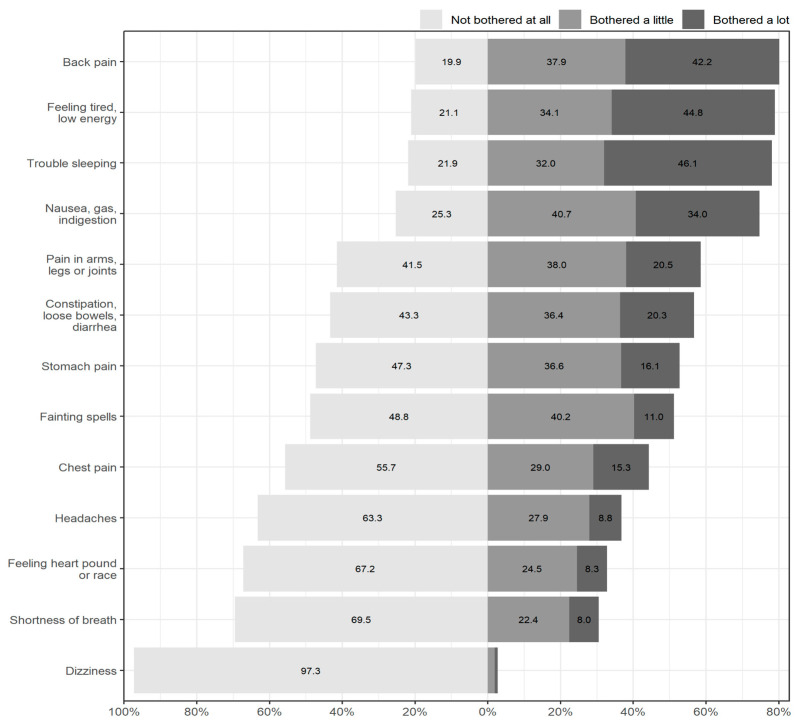
Prevalence of Somatic Symptoms.

Figure 1 shows response proportions for the 13 somatic symptoms included in the study in decreasing order according to prevalence. The most frequently observed symptoms, with an incidence higher than 70% of the total sample, were back pain *p* = 0.801 (95% CI: 0.787–0.815), tiredness *p* = 0.789 (95% CI: 0.775–0.803), trouble sleeping *p* = 0.781 (95% CI: 0.767–0.795), and gastrointestinal complications such as nausea, gas or indigestion *p* = 0.747 (95% CI: 0.732–0.762). Almost half of the sample reported pain in the chest *p* = 0.443 (95% CI: 0.425–0.460), arms, legs or joints *p* = 0.585 (95% CI: 0.568–0.602), stomach pain *p* = 0.527 (95% CI: 0.510–0.544) along with constipation, loose bowels, or diarrhea *p* = 0.567 (95% CI: 0.550–0.584), and fainting spells *p* = 0.512 (95% CI: 0.495–0.529). Less than 40% experienced headaches *p* = 0.367 (95% CI: 0.350–0.384), feelings of heart race *p* = 0.328 (95% CI: 0.311–0.344) or shortness of breath *p* = 0.305 (95% CI: 0.289–0.320 were less frequent. In addition, almost no one had feelings of dizziness *p* = 0.027 (95% CI: 0.022–0.033).

### 3.2. Severity of Somatic Symtpoms

Almost half of the participants (48.14%) reported a moderate-to-severe intensity of somatic symptoms. By examining sociodemographic strata, women showed higher severity of symptoms compared to men, with more than double the percentage of cases in the severe category. Students who were working while studying showed similar patterns of severity compared with the unemployed ones. Regarding relationship status, participants who were in a relationship had a slightly lesser number of respondents in the moderate-to-severe categories than the single ones. No clear pattern was found regarding body mass index. COVID-19-related variables denote that the students who are part of the risk group for COVID-19 have higher severity than students who are not considered at risk. Having a relative diagnosed with COVID-19 or who lost their job due to the pandemic implied a higher severity of somatic symptoms. Specific percentages are shown in Table 1.

### 3.3. Factors Associated with Somatic Symtpom Severity

Severity categories were dichotomized to a two-level variable with none-to-low, and moderate-to-severe levels. This indicator was used as a dependent variable in a general linear model. The results are shown in Table 2, and no evidence of severe multicollinearity was found (Variance Inflation Factor < 10). Regarding sociodemographic variables, strong differences were found regarding sex ($OR_{adj}$ = 2.484), suggesting that women have approximately 150% more odds of experiencing moderate-to-severe somatic symptoms in comparison to men. Likewise, we found that relationship status was a statistically significant predictor of somatic symptom severity ($OR_{adj}$ = 1.422); participants in a relationship had 40% more chances of experiencing moderate-to-severe somatic symptoms. Daily hours of sleep had a statistically significant negative relationship with somatic symptoms. Lastly, being part of the COVID-19 risk group ($OR_{adj}$ = 1.298), having a relative diagnosed with COVID-19 ($OR_{adj}$ = 1.200) or who works at a position exposed to the disease ($OR_{adj}$ = 1.508) increased the odds of more severe somatic symptoms by 20% to 50%, approximately. No differences were found according to body mass index, employment status, age, nor the number of semesters concluded.

With respect to psychological variables, a moderate negative relationship with somatic symptom severity was found for satisfaction with life, resilience, and perceived social support from family, friends, or significant other, on bivariate analyses. However, after controlling for the effect of other variables, all these relationships were insignificant. On the contrary, academic self-efficacy ($OR_{adj}$ = 0.857) was the only statistically significant predictor of somatic symptom severity after controlling for the effect of other variables, with a negative relationship which indicates that the odds of moderate-to-severe somatic symptoms are 14% less likely in those with higher academic self-efficacy.

Regarding psychopathological variables, strong positive bivariate relationships were found between somatic symptom severity and the mental health conditions depression, anxiety, and stress, on bivariate analyses. After adjusting the effect of the other variables, only anxiety ($OR_{adj}$ = 3.592) had a statistically significant positive relationship with somatic symptom severity, which indicates that the odds of moderate-to-severe somatic symptoms are 259% more likely in those with higher anxiety.

## 4. Discussion

The present study aimed to analyze the prevalence of somatic symptoms in university students, considering the respective level of severity. Additionally, the second objective of the study was to analyze the relationship between somatic symptom severity and various psychological, psychopathological, and sociodemographic variables that have been a focus of study in mental health research carried out in the context of the COVID-19 pandemic. Our main results regarding the prevalence of somatic symptoms show that more than half of the participants report having experienced psychosomatic difficulties, mainly back pain, feeling constantly tired, trouble sleeping, gastrointestinal discomfort, and pain in the arms or joints. As an alarming note, almost 45% of the participants had moderate-to-severe discomfort, a percentage that varies according to sociodemographic variables. Contemporary literature shows that somatizations manifest with a high prevalence in university students [16,39]. This prevalence may be associated with genetic and environmental factors, as well as life experiences [40]; in the case of university students, an increase in prevalence is attributed because academic demands represent potential stressors that evoke a series of bodily ailments such as headaches, back pain, joint pain, or limb pain, as well as breathing difficulties or an increase in heart rate [41]. In addition, some studies have found a strong relationship between the increase in somatic symptom severity and comorbidity with other mental health conditions such as depression, anxiety, and stress in university students [42,43]. This relationship is particularly important, since in the context of the COVID-19 pandemic, an increase in these mental health conditions has been reported internationally [6,44,45]. In this sense, given the strong relationship between somatizations and mental health conditions, an increase in the prevalence of somatic manifestations is expected.

By analyzing sociodemographic factors related to somatic symptom severity, strong differences between men and women were still found after controlling for the effect of other variables, which indicated that women experience more severe somatic symptoms. Current literature on mental health during the COVID-19 pandemic shows similar results, suggesting that women had a higher severity of somatic symptoms as well as a higher rate of other mental health conditions such as depression, anxiety, or stress [46,47,48,49,50]. Explanations could be linked to social studies in Latin American countries that found an increase in domestic violence against women during the pandemic [51,52,53], as well as an increasing withdrawal of their occupations to take on household chores [54]. In addition, our results suggest that being in a relationship is considered as a risk factor towards experiencing more severe somatic symptoms; previous studies have found mixed results regarding this issue, since some indicated no particular effect of relationship status on somatic symptoms [55] and others suggested that being single may be a protective factor as it reduces the likelihood of getting infected [56]. Daily hours of sleep was a strong predictor of somatic symptom severity, an expected result since difficulties sleeping are in fact manifestations of the somatic syndrome [57]; besides, the COVID-19 pandemic brought multiple stressors that affected the sleep quality of most people [58]. No significant relationships were found regarding age, employment status, body mass index, or the number of semesters completed by the participants.

Regarding the COVID-19 specific variables, we found that being part of a risk group towards COVID-19 was linked to a higher severity of somatic symptoms, a result that is consistent with current research and explained through the constant mental health burden experienced for being vulnerable to the disease [59]. Concerning family, we found that having a family member diagnosed with COVID-19 is a risk factor for experiencing more severe somatic symptoms. Lorenzo and Carrisi [60] suggested that having a relative exposed to the disease implies that the co-residents are also extremely exposed to an infection. For instance, it has been found that relatives of healthcare professionals are subject to stress, isolation, and emotional distress during the pandemic [61].

By analyzing psychological protective factors, we found a negative relationship with somatic symptom severity for perceived social support, resilience, and life satisfaction, in bivariate analyses. These variables are constantly linked to a lesser severity of mental health conditions and somatic symptoms [22,55,62,63]. In fact, having social support, a strong resilience trait, and a positive perception towards life allow students to better cope with stressors, and a better management of negative thoughts and emotional regulation [9,64]. In this sense, these characteristics allow university students to better adapt to the COVID-19 pandemic context; thus, increasing overall well-being and preventing mental health conditions such as depression, anxiety, and stress [65,66,67]. Nonetheless, most of these studies have not considered adding multiple psychological, psychopathological, or demographic factors as covariates as an intent to control the effect of other potential predictors. We found no statistically significant relationship between somatic symptom severity and the support from family, friends, or a significant other, resilience, and life satisfaction after controlling for the effect of other variables, which suggests that other factors may have a higher impact on somatic symptom severity. On the contrary, we found that higher academic self-efficacy was a statistically significant predictor of a lower somatic symptom severity, even after controlling for the effect of all other variables. This negative relationship has been previously found in the literature [68]. Similarly, Meyer et al. [69] found that having a high academic self-efficacy allowed students to better cope with stressors during the pandemic and to effectively deal with academic tasks.

With respect to psychopathological variables, we found a strong relationship between somatic symptom severity and the mental health conditions depression, anxiety, and stress, which is consistent with contemporary studies [25,70]. Nevertheless, after controlling for the effect of most variables, depression and stress showed a non-statistically significant relationship with somatic symptom severity. This may occur due to the statistical control of all other variables, mainly the strong effect shown by anxiety, which may imply a potential mediating role previously tested in the literature [71]. We found that anxiety had the highest effect size among all variables considered in the model, indicating a significant comorbidity with somatic symptoms. In this sense, higher levels of anxiety are considered risk factors to experience a more severe somatic syndrome. Similar results were found in current literature; for instance, Shevlin et al. [72] found a positive relationship between anxiety towards the COVID-19 and somatic symptoms with a stronger link experienced in gastrointestinal discomfort and fatigue, which were two of the most experienced symptoms on our current sample. Similar results were found in diverse contexts such as China [73], and Norway [74]. The explanation behind this finding is that a pathological experience of somatic symptoms is not necessarily confined to physical complaints, but rather entails psychological and behavioral aspects such as health anxiety and constant checking behaviors [75]. Even though anxiety acts as an adaptive response that allows the subject to avoid potential harm, it may be a factor in provoking or maintaining medical conditions, such as somatic distress [76]. For this reason, interventions on anxiety have been previously recommended as an alternative to deal with somatic symptom severity [77].

### Limitations

Is important to recognize that our study has some limitations. Our sampling procedures do not consider a randomized selection of participants. In this sense, results cannot be generalized to a wider population, only to participants of the current study and potentially to persons who have similar sociodemographic characteristics as the ones included in the present study, such as university students from Lima, Peru. In this sense, external validity of the current study may be limited, and further studies are required on similar and different contexts to contrast our results. Nevertheless, somatic symptoms have been an overlooked topic on studies regarding the COVID-19 pandemic [15], and our results can be considered as an exploratory contribution to the literature.

Additionally, since we used secondary data, we were limited to use only the available variables and could not consider the inclusion of other potentially relevant variables related to somatic symptom severity in the study. As a consequence, we can only draw conclusions about the relationships between the included predictors, limiting other possible interactions that could have influenced the results and could not be controlled; for instance, if a participant was taking any medication or drug that causes effects or side-effects related to the somatization symptoms measured by the PHQ-15 [78] or who has attended to psychotherapeutic interventions [79]. Despite these limitations, our findings show strong insights on the demographic, psychological, and psychopathological factors that influence the severity of somatic symptoms in university students during the COVID-19 pandemic.

## 5. Conclusions

We found that anxiety was the strongest predictor among all variables considered. With respect to psychological factors, academic self-efficacy was a significant predictor of somatic symptom severity, which denotes the importance of adequately addressing academic demands for university students. On sociodemographic variables, we found significant differences regarding sex, relationship status, and daily hours of sleep, as well as a more severe experience of somatic symptoms for participants who are part of the COVID-19 risk group, who had a relative who worked at a job which exposed them to the COVID-19 or who was already diagnosed with it. To reduce somatic symptom severity, anxiety-related interventions may have the stronger impact, followed by enhancing academic self-efficacy as well as working on promoting healthy sleep schedules. Special emphasis should be given to women, since our results as well as international findings indicate that they experience the most severe somatic symptoms and other mental health conditions in comparison to men.

## Figures and Tables

**Table 1:** Severity of Somatic Symptoms by Sociodemographic Groups.

Strata	Level	*n*	Severity			
			None	Low	Moderate	Severe
Total		3218	18.52	33.34	30.27	17.87
Sex	Man	1028	32.00	37.55	22.47	7.98
	Woman	2190	12.19	31.37	33.93	22.51
Employment status	Unemployed	2588	18.78	34.16	29.64	17.43
	Employed	630	17.46	30.00	32.86	19.68
Relationship status	Not in a relationship	2196	19.35	34.65	29.87	16.12
	In a relationship	1022	16.73	30.53	31.12	21.62
Body mass Index	<18.5	197	22.84	25.38	35.03	16.75
	≥18.5	2247	18.20	33.91	30.35	17.53
	≥25	635	17.01	33.70	29.13	20.16
	≥30	139	24.46	33.81	27.34	14.39
COVID-19 risk group	No	2568	19.94	34.00	29.40	16.67
	Yes	650	12.92	30.77	33.69	22.62
Relative diagnosed with COVID-19	No	1321	22.18	35.05	27.48	15.29
	Yes	1897	15.97	32.16	32.21	19.66
Relative exposed to COVID-19 at work	No	2337	20.54	34.49	28.63	16.35
	Yes	881	13.17	30.31	34.62	21.91

**Table 2:** Predictors of Somatic Symptom Severity.

Variable	*OR* (95% CI)	*OR_adj_* (95% CI)
Depression	2.93 *** (2.68–3.22)	0.97 (0.80–1.17)
Anxiety	4.21 *** (3.78–4.72)	3.59 *** (2.72–4.75)
Stress	3.89 *** (3.50–4.34)	1.11 (0.84–1.48)
PSS–Significant other	0.72 *** (0.67–0.77)	0.89 (0.75–1.06)
PSS–Family	0.63 *** (0.59–0.68)	0.92 (0.80–1.06)
PSS–Friends	0.76 *** (0.71–0.82)	1.10 (0.97–1.26)
Resilience	0.61 *** (0.57–0.66)	1.12 (0.99–1.27)
Satisfaction with life	0.64 *** (0.60–0.69)	0.99 (0.88–1.13)
Academic self-efficacy	0.63 *** (0.59–0.68)	0.86 ** (0.76–0.96)
Sex	2.96 *** (2.53–3.47)	2.48 *** (2.05–3.02)
Age	1.01 (0.97–1.05)	1.04 (0.96–1.12)
Semesters	1.02 (0.99–1.04)	1.00 (0.95–1.04)
Employment status	1.25 * (1.05–1.48)	1.24 (0.98–1.56)
Relationship status	1.31 *** (1.13–1.52)	1.42 ** (1.16–1.75)
Sleep hours	0.79 *** (0.74–0.83)	0.86 *** (0.81–0.92)
Body Mass Index	0.99 (0.97–1.01)	0.98 (0.95–1.00)
COVID-19 risk group	1.51 *** (1.27–1.80)	1.30 * (1.04–1.62)
Relative diagnosed with COVID-19	1.44 *** (1.25–1.66)	1.20 * (1.01–1.43)
Relative exposed to COVID-19 at work	1.59 *** (1.36–1.86)	1.51 *** (1.25–1.83)

Note. PSS = Perceived Social Support, OR = Odds ratios, $OR_{adj}$ = adjusted odds ratios, CI = confidence intervals, SE = standard error, z = z value, *p* = *p* value. OR represent the bivariate relationship between each predictor and the somatic symptom severity. Units for depression to academic self-efficacy are standardized factor scores obtained from the measurement models. Age is expressed in years. Semesters are expressed in number of semesters completed up to the date of the evaluation. Sleep hours are presented in daily hours of sleep. Body Mass Index is presented as a ratio between an adult weight in kilograms by their height in meters squared. * *p* < 0.05, ** *p* < 0.01, *** *p* < 0.001.

## Data Availability

The data that support the findings of this study are available from the Faculty of Psychology of the Universidad de Lima, but restrictions apply to the availability of these data, which were used under license for the current study, and thus are not publicly available. Data are however available from the authors upon reasonable request and with the permission of the Faculty of Psychology of the Universidad de Lima.
